# Supplementary material for: Quantifying the role of variability in future intensification of heat extremes
Source: Nat Commun. 2022 Dec 24;13:7930. doi: 10.1038/s41467-022-35571-0 (PMC9790021; doi:10.1038/s41467-022-35571-0)
Supplement: Supplementary file 1 — Supplementary Information [file 41467_2022_35571_MOESM1_ESM.pdf]

Supplementary Information for

Quantifying the role of variability in future intensification of heat extremes

Claudia Simolo*, Susanna Corti*

* Correspondence: c.simolo@isac.cnr.it or s.corti@isac.cnr.it.

Contents

- Supplementary Tables 1 to 4
- Supplementary Figures 1 to 13
- Supplementary Note

Supplementary Table 1. **Model Outputs**

GCM (*Institution*)	Run	#lon x #lat	$\langle t \rangle_{+2K}$	$\langle t \rangle_{+3K}$
ACCESS-CM2 (*CSIRO-ARCCSS*)	r1i1p1f1	192 x 144	2038	2055
ACCESS-ESM1-5 (*CSIRO*)	r1i1p1f1	192 x 145	2039	2060
BCC-CSM2-MR (*BCC*)	r1i1p1f1	320 x 160	2043	2065
CNRM-CM6-1 (*CNRM-CERFACS*)	r1i1p1f2	256 x 128	2040	2058
CNRM-ESM2-1 (*CNRM-CERFACS*)	r1i1p1f2	256 x 128	2045	2064
CanESM5 (*CCCma*)	r1i1p1f1	128 x 64	2022	2040
EC-Earth3 (*EC-Earth-Consortium*)	r4i1p1f1	512 x 256	2036	2055
EC-Earth3-Veg (*EC-Earth-Consortium*)	r4i1p1f1	512 x 256	2034	2055
GFDL-ESM4 (*NOAA-GFDL*)	r1i1p1f1	288 x 180	2053	2076
HadGEM3-GC31-LL (*MOHC*)	r1i1p1f3	192 x 144	2030	2047
INM-CM4-8 (*INM*)	r1i1p1f1	180 x 120	2046	2069
INM-CM5-0 (*INM*)	r1i1p1f1	180 x 120	2046	2074
IPSL-CM6A-LR (*IPSL*)	r1i1p1f1	144 x 143	2034	2050
MIROC6 (*MIROC*)	r1i1p1f1	256 x 128	2053	2076
MPI-ESM1-2-HR (*DKRZ*)	r1i1p1f1	384 x 192	2049	2073
MPI-ESM1-2-LR (*MPI-M*)	r1i1p1f1	192 x 96	2049	2071
MRI-ESM2-0 (*MRI*)	r1i1p1f1	320 x 160	2038	2064
NESM3 (*NUIST*)	r1i1p1f1	192 x 96	2034	2054
NorESM2-MM (*NCC*)	r1i1p1f1	288 x 192	2054	2076
UKESM1-0-LL (*MOHC*)	r1i1p1f2	192 x 144	2031	2046

Details of CMIP6 simulations: model name (institution), probed run, horizontal resolution and approximate timings of +2K and +3K warming under the high-end scenario SSP5-8.5. In the median of model results, $\langle t \rangle_{+2K}$ $\sim$2040 and $\langle t \rangle_{+3K}$ $\sim$2062. Higher warming levels are not passed by the end of the century in several SSP5-8.5 simulations.

Supplementary Table 2. **Summary of future changes**

Observable	*Region*	+2K	+3K
ΔTXx $[K]$	*Globe*	1.9 (1.7-2.1)	2.8 (2.6-3.1)
	Global land	2.6 (2.1-3.1)	3.9 (3.4-4.6)
	EMD (JJA)	3.5 (1.9-4.2)	4.9 (4.1-6.2)
	CSA (SON)	3.4 (2.5-4.6)	5.0 (4.1-6.7)
ΔTNn $[K]$	*Globe*	2.6 (2.2-2.8)	3.8 (3.3-4.0)
	Global land	3.4 (2.9-3.8)	5.1 (4.2-5.6)
	PAr (DJF)	7.9 (4.7-11.1)	12.5 (8.6-16.0)
	MHL (DJF)	5.2 (4.0-6.3)	7.6 (5.6-8.8)
	SOc (JJA)	3.1 (0.7-5.2)	4.6 (1.1-7.5)
$FCEP$-99th	*Globe*	24 (15-37)	40 (32-55)
	Global land	7 (5-11)	14 (12-21)
	TOs	48 (24-68)	74 (53-89)
	TLs	11 (8-18)	23 (18-35)
$FCEP$-99.9^{th}	*Globe*	126 (44-230)	265 (142-419)
	Global land	19 (13-34)	53 (47-62)
	TOs	294 (67-486)	583 (250-785)
	TLs	33 (20-61)	97 (61-171)

Global and hotspot changes in the magnitude of the hottest days (ΔTXx) and the coldest nights (ΔTNn) and in the probability of hot ($FCEP$-99th) and very hot days ($FCEP$-99.9^{th}). Changes are given by the median of the 20 model results and their total spread (in brackets), at +2K and +3K warming under the high-end scenario SSP5-8.5. Regions are defined in the main text (see the *Hotspot* section), except for *SOc* which denotes the Southern Ocean band (50S-70S). Changes in probability are expressed in fractional terms relative to the early industrial levels (as in Fig.6b of the main text).

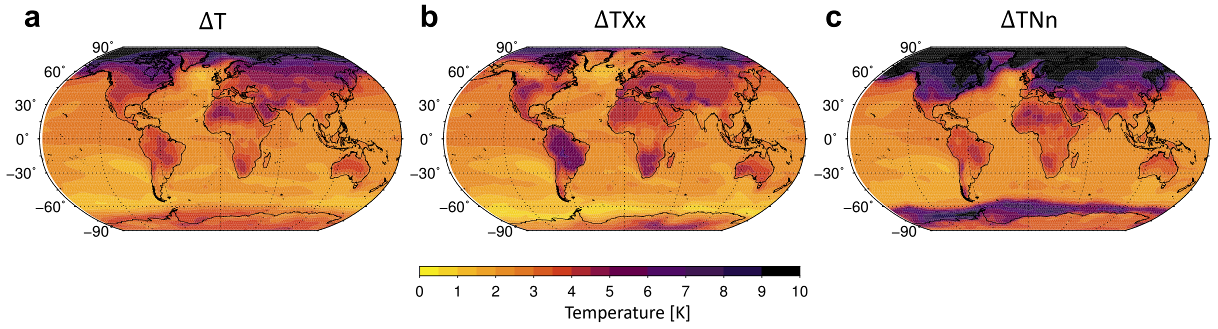

Supplementary Figure 1. **Annual changes at +3K warming.** Multimodel changes in (**a**) annual mean temperatures T, (**b**) the hottest days TXx and (**c**) the coldest nights TNn of the year.

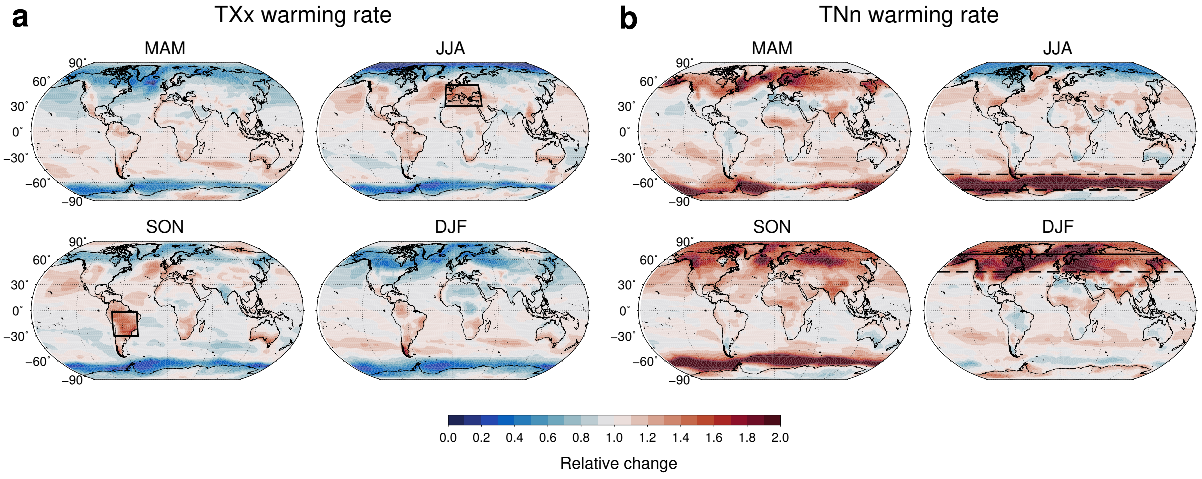

Supplementary Figure 2. **Seasonal extremes at +3K warming.** Multimodel warming rates of (**a**) the hottest days TXx and (**b**) the coldest nights TNn of every season. MAM denotes March-April-May, JJA June-July-August, SON September-October-November and DJF December-January-February.

Supplementary Note. Analytical relations between moments and extremes

Temperature extremes may increase in both magnitude and frequency with greenhouse warming. Changes in the magnitude of fixed-frequency events strictly depend on the type of changes undergone by the underlying distribution, irrespective of its native shape. If the latter is preserved under an upward shift, then the distribution mean and extremes increase by the same amount, keeping their distance fixed. Instead, changes in the higher order moments can alter this distance and lead to additional changes in the extremes. This is schematically shown in Supplementary Fig.3a, where pure Gaussian temperatures (i.e., with neutral skew) undergo a decrease in variability concurrently with an upward shift. Hot extremes (x_+) are seen to warm slower than the mean (μ), whereas cold extremes (x_-) warm faster. The opposite holds if variability is increased.

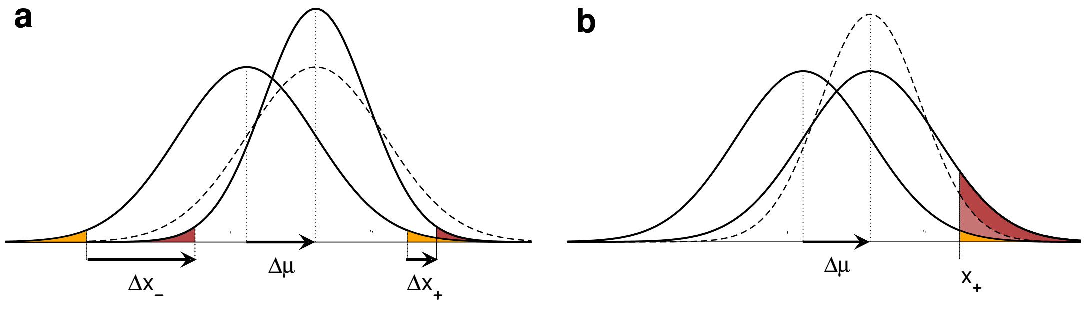

Supplementary Figure 3. **Conceptual picture of distribution changes.** Effects of a simultaneous change in the distribution mean (increase) and standard deviation (decrease) on (**a**) the magnitude and (**b**) the frequency of the extremes, assuming Gaussian temperatures. In either case changes in hot extremes are reduced by a decrease in variability, as compared to a rigid shift. Shaded areas denote exceedance probabilities.

For normal (or quasi-normal) temperatures, changes in the magnitude of the extremes ($x_\pm$) arising from a simultaneous change in the mean μ and standard deviation σ of the underlying distribution can be written as

$$(x_\pm(w) - \mu(w))^{-1}\frac{d}{dw}(x_\pm(w) - \mu(w)) = \sigma(w)^{-1}\frac{d}{dw}\sigma(w) \quad (1)$$

where w is a given external variable (e.g., time or warming), $x_\pm$ are some large quantiles with fixed probability and the distribution parameters μ and σ depend continuously on w. Supplementary Equation (1) shows that the fractional change in the extreme-to-mean distance equals the fractional change in standard deviation. Thereby, decrease

in standard deviation slows down hot extremes relative to the mean and further accelerates cold extremes ($x_- < \mu < x_+$ by definition, for any w). Supplementary Equation (1) can be easily obtained by the condition $dP(x \geq x_+)/dw = 0$ for hot extremes x_+ together with the analogous condition for cold extremes x_-, using the Gaussian density $f = \exp(z^{-2})/\sigma\sqrt{2\pi},\ z = (x - \mu)/\sigma\sqrt{2}$ as an integrand. The above equation equally applies to skewed distributions as long as changes in skewness (and kurtosis) remain negligible. If they do not, additional terms appear in the right-hand side and account for higher than second order changes as a group (denoted $h.o.$ in Equation (1) of the main text). These changes may either reinforce or lessen the effects of changes in variability, contributing to the excess warming of the extremes relative to the mean. For example, if skewness increases, the high-end tail gets longer and hot extremes warm faster, moving away from the mean.

Conversely, changes in the frequency of fixed-magnitude events sharply depend on the native structure of thermal distributions, particularly on variability. In the case, again, of a Gaussian distribution, with constant standard deviation σ, the rate of change in the probability of exceeding a large quantile x_+ (i.e., $x_+ = t\sigma$ with t fixed) is given by

$$\frac{dP(x \geq x_+; w)}{dw} = \frac{v}{\sigma\sqrt{2\pi}} \exp\left[-\left(\frac{t}{\sqrt{2}} - \frac{vw}{\sigma\sqrt{2}}\right)^2\right], \tag{2}$$

where, without loss of generality, we assumed a constant shifting velocity v, i.e., $\mu(w) = vw$ with $\mu(w_0) = 0$ for some initial condition w_0. As is clear from Supplementary Equation (2), for any pair of constant parameters v and σ, probability rates of change increase with increasing warming w –the more the higher is v and the lower is σ–, as long as vw stays below $t\sigma$. If this limit is exceeded for some w, the extremes become as common as the mean. With the above assumptions, probability rates depend on v and σ only through their ratio v/σ (the standard shift velocity). Thus, halving σ is the same as doubling v and, because of nonlinearity, this leads to a more than doubling of probability rates. As a result, changes in exceedance probabilities are highly sensitive to the native width of thermal distributions, and even little underestimation of the latter may cause a large overestimation of future probabilities. For example, based on Supplementary Equation (2), it can be shown that a 20% error on native σ may translate into a $\sim$40-60% error on probability changes (depending on σ and the level of warming).

Actually, the above ideal Gaussian shift may oversimplify real world conditions, because temperature anomalies are often skewed while both variability and the shift velocity may change with the global level of warming. As compared to symmetric distributions, left skew tends to sharpen rates of change in the warm tail probabilities and to flatten those in the cold ones (viceversa right skew). Furthermore, changes in the second and higher order moments can modify the probability rates of change given by Supplementary Equation (2) and lead to additional amplification of the frequency of the extremes as well as to its partial suppression, as seen in Supplementary Fig.3b.

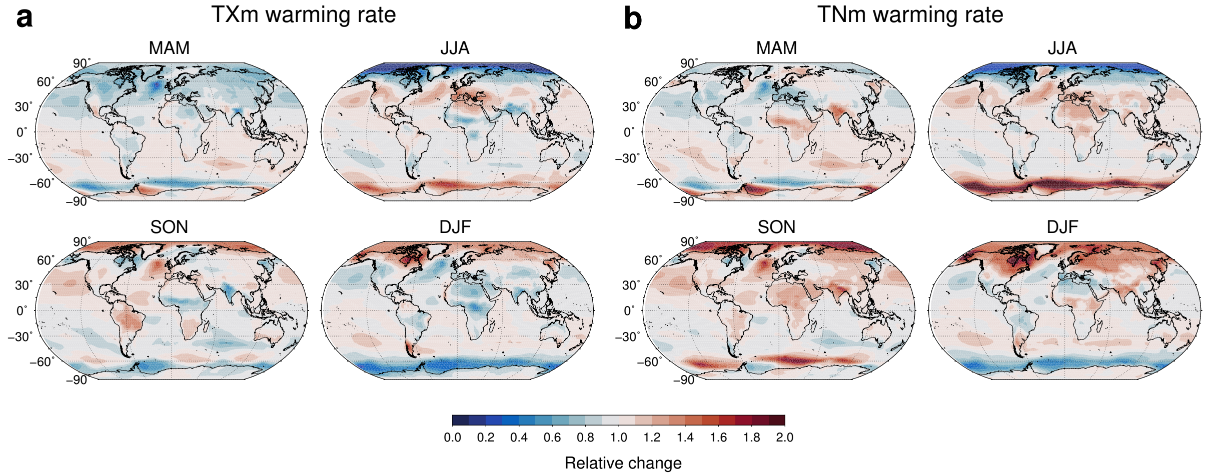

Supplementary Figure 4. **Seasonal mean changes.** Multimodel seasonal warming rates of (**a**) the daytime mean TXm and (**b**) the nighttime mean TNm, at +2K warming. MAM denotes March-April-May, JJA June-July-August, SON September-October-November and DJF December-January-February.

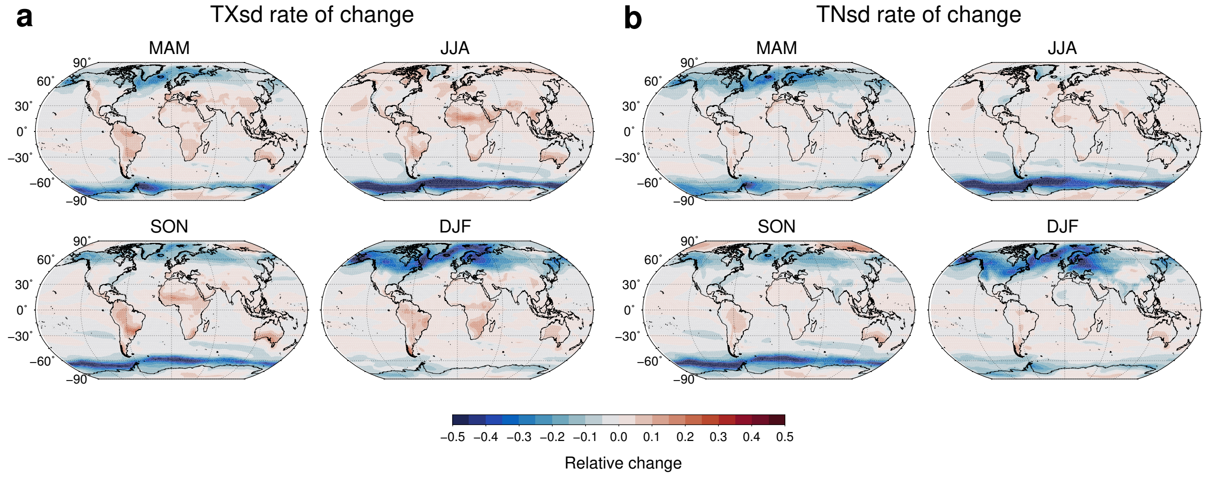

Supplementary Figure 5. **Variability changes at +3K warming.** Multimodel seasonal rates of change in standard deviation (**a**) $TXsd$ and (**b**) $TNsd$. MAM denotes March-April-May, JJA June-July-August, SON September-October-November and DJF December-January-February.

Supplementary Table 3. **Correlations between changes in the extremes and variability**

	MAM	JJA	SON	DJF
$r(\Delta_T(TXx - TXm),\, \Delta_T TXsd)$	0.88	0.91	0.86	0.88
$r(\Delta_T(TNn - TNm),\, \Delta_T TNsd)$	-0.94	-0.94	-0.95	-0.90

Global area-weighted correlations (Pearson's r) of the excess rates of the hottest days TXx and the coldest nights TNn with the rates of change in standard deviation $TXsd$ and $TNsd$ respectively, in every season. Namely, $\Delta_T(TXx - TXm) = (\Delta TXx - \Delta TXm)/\Delta T$ and $\Delta_T TXsd = \Delta TXsd/\Delta T$ where ΔT is the change in annual mean temperatures. Similarly for TN anomalies. Results are based on multimodel quantities over the common grid, at +2K warming.

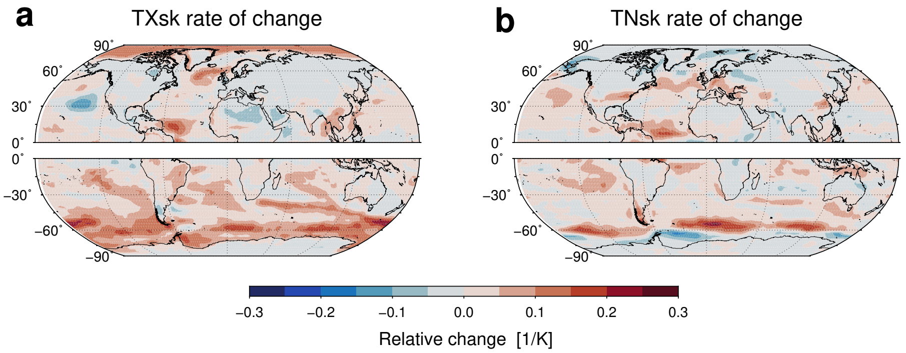

Supplementary Figure 6. **Skewness changes.** Multimodel rates of change of (**a**) TX and (**b**) TN skewness in the summer and the winter hemispheres respectively, at +2K warming.

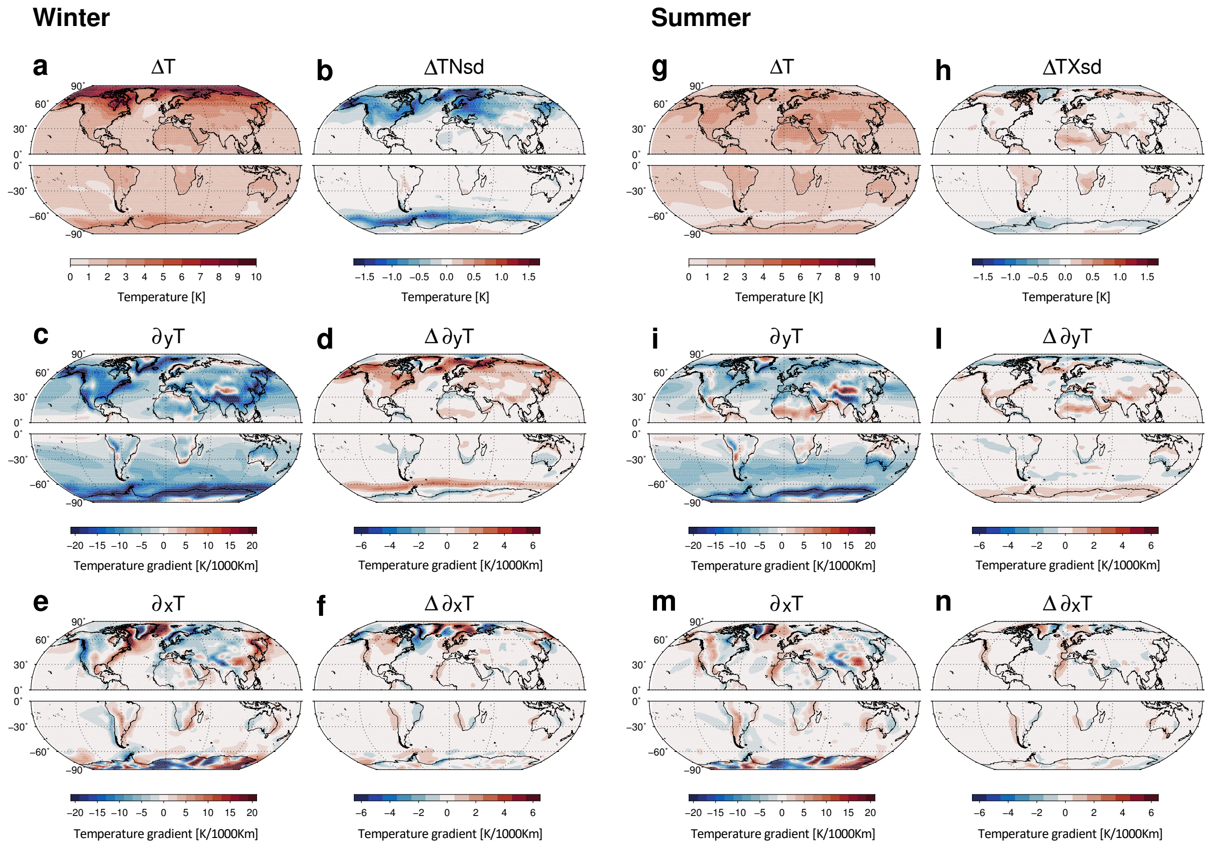

Supplementary Figure 7. **Multimodel changes in horizontal temperature gradients and variability. a**,**b** Winter changes in mean temperatures T and nighttime standard deviation $TNsd$ respectively, at +2K warming. **c**,**d** Winter early-industrial meridional temperature gradient $\partial_y T = \partial T/\partial|y|$ and its +2K changes, respectively. **e**,**f** Same as **c**,**d** but for the zonal gradient $\partial_x T = \partial T/\partial x$. **g-n** Same as **a-f** but for the summer hemispheres and with daytime standard deviation $TXsd$ (**h**) in place of $TNsd$. Globally, grid-point $TNsd$ and $TXsd$ changes positively correlate with those in the meridional gradient during winter and summer respectively (with r roughly ranging from 0.4 to 0.5 in both cases). Correlations are taken with gradient changes multiplied by the sign of the early industrial gradient, such that the latter is weakened (reinforced) by negative (positive) changes highlighting monotonic relationships with changes in variability.

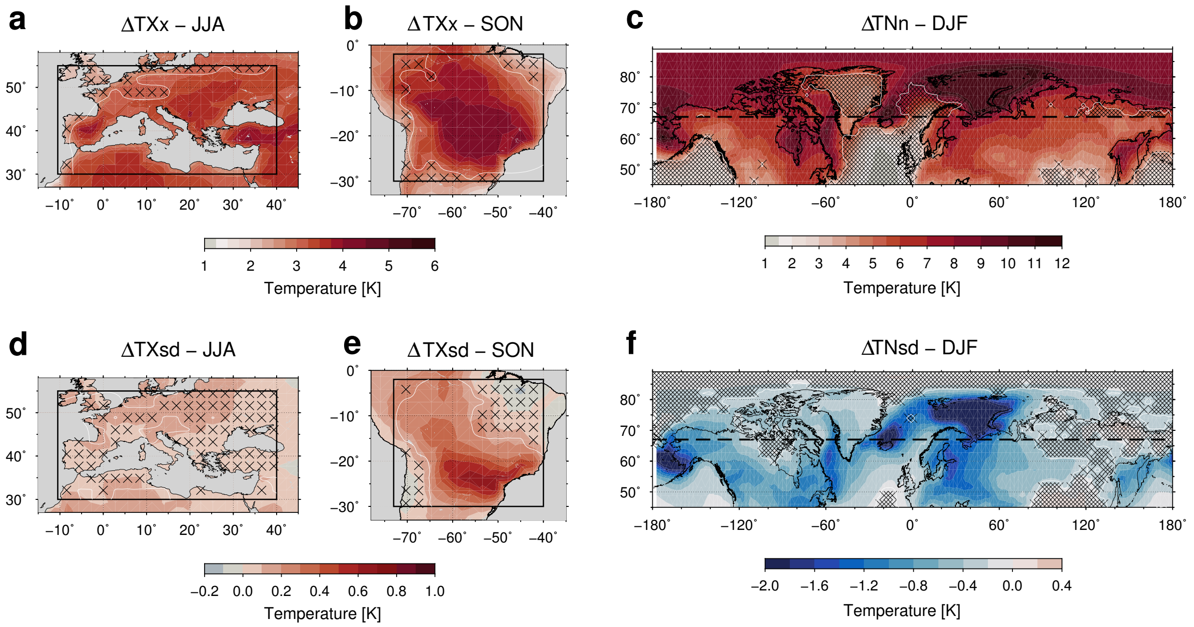

Supplementary Figure 8. **Robustness of total regional changes at +2K warming.** **a**,**b** Multimodel changes in the hottest days TXx over the Euro-Mediterranean (EMD) zone and Central South America (CSA) in June-July-August (JJA) and September-October-November (SON) respectively. Cross marks denote land grid points where less than 15 models out of 20 simulate TXx warming above the +2.5K regional background. **c** Multimodel changes in the coldest nights TNn over the Pan-Arctic (PAr) zone and mid-to-high latitudes (MHL) in December-January-February (DJF), including all land and ocean north of 45N (the Arctic Circle is dashed). Hatching denotes areas where less than 15 models simulate TNn warming above the regional background ($\sim$+5K and $\sim$+3K in the PAr and the MHL region respectively). The area fraction where warming of the extremes is robustly predicted to exceed the regional background amounts to $\sim$80% in the EMD, the CSA and the PAr zone and to over 70% in the MHL zone. **d-f** Multimodel changes in standard deviation (**d**,**e**) $TXsd$ and (**f**) $TNsd$ restricted to regions and seasons as above. (**d**,**e**) Cross marks denote land grid points where less than 15 models simulate positive changes, whereas (**f**) hatching denotes areas where less than 15 models simulate negative changes. The area fractions robust to either (**d**,**e**) positive or (**f**) negative changes amount to about 30% (EMD), 70% (CSA and PAr) and 90% (MHL). Results are based on the high-end scenario SSP5-8.5.

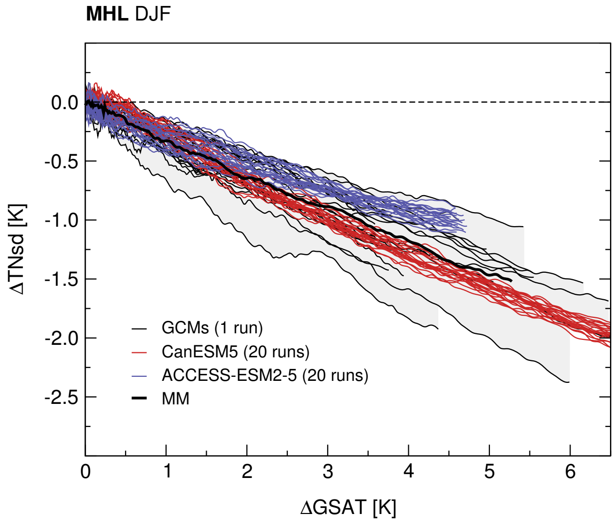

Supplementary Figure 9. **Intermodel versus intramodel spreads.** Projected changes in standard deviation $TNsd$ at mid-to-high latitudes (MHL) in December-January-February (DJF), as a function of changes in global-mean surface air temperature (GSAT) under the high-end scenario SSP5-8.5. The total spread across the 20 CMIP6 models (one run, Supplementary Table 1) is compared with the individual spreads across 20 realizations (r1-r20) of two models, i.e., CanEMS5 and ACCESS-ESM2-5. The latter have been chosen for both their run multiplicity and their rather different projections of $TNsd$ changes, either below or above the multimodel mean (MM). Intramodel spreads are seen to lie well within the broader intermodel spread, thus justifying the use of the latter as a first order estimate of projection uncertainties in temperature related variables. In addition, all models and runs clearly suggest a near-linear behavior of variability changes with greenhouse warming.

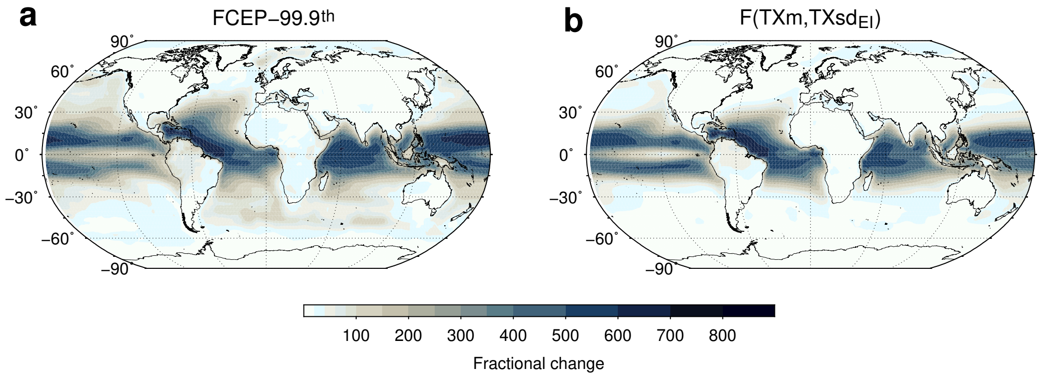

Supplementary Figure 10. **Probabilities of very hot days.** **a**,**b** Multimodel fractional changes in very-hot day probabilities at +2K warming and their theoretical representation by Equation (2) (as in Fig.7a of the main text), respectively.

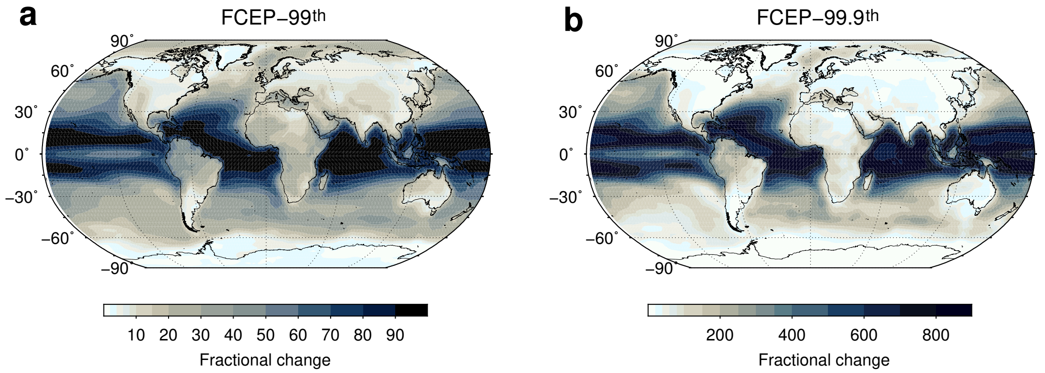

Supplementary Figure 11. **Hot event probabilities at +3K warming.** Multimodel fractional changes in the probability of (**a**) hot and (**b**) very hot days.

Supplementary Table 4. **Correlations between probability changes and variability**

	+2K	+3K
$\rho(FCEP\text{-}99^{th},\, TXsd_{EI})$	-0.90	-0.90
$\rho(FCEP\text{-}99.9^{th},\, TXsd_{EI})$	-0.89	-0.89
$\rho(FCEP\text{-}99^{th},\, \Delta_{EI}TXsd)$	0.45	0.45
$\rho(FCEP\text{-}99.9^{th},\, \Delta_{EI}TXsd)$	0.43	0.42

Global rank correlations (Spearman's ρ) of fractional changes in hot ($FCEP$-99th) and very-hot day probabilities ($FCEP$-99.9^{th}) with standard deviation over the early industrial era $TXsd_{EI}$ and with its fractional changes (i.e., $\Delta_{EI}TXsd = \Delta TXsd/TXsd_{EI}$), at +2K and +3K warming. Correlations are based on multimodel results over the common grid and are highly significant (p-values < 0.01, using a t-distribution).

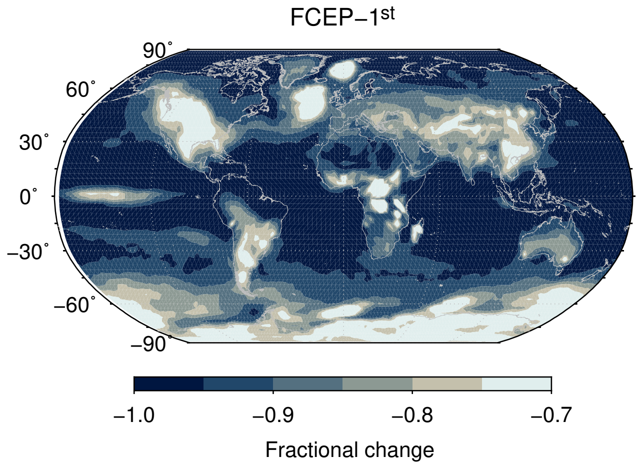

Supplementary Figure 12. **Cold night probabilities.** Multimodel fractional changes in the probability of TN anomalies colder than the early-industrial 1st percentile ($FCEP$-1st) at +2K warming.

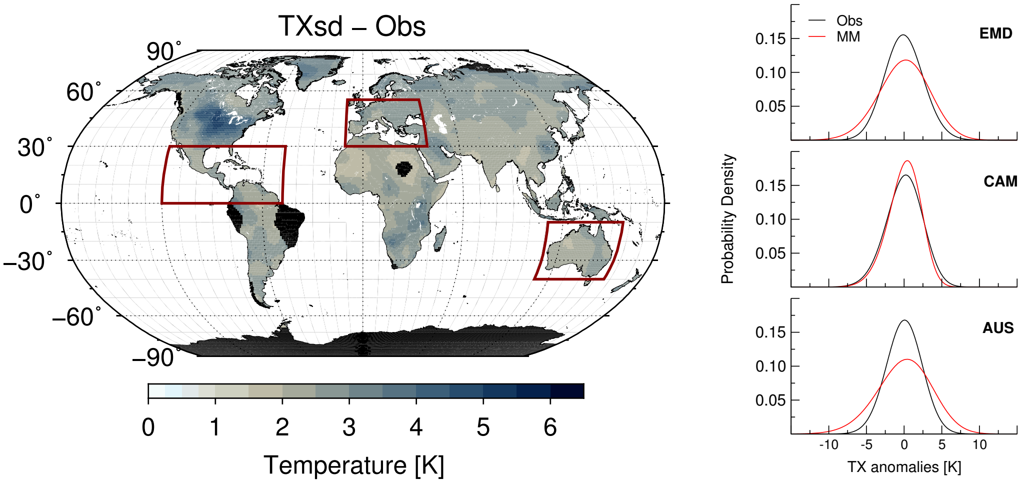

Supplementary Figure 13. **Insights from observations.** Global map of observed historical standard deviation $TXsd$ over land from Berkeley Earth data (spanning 1880 to the present). Grid point $TXsd$ are obtained, like for models, from daily TX anomalies (see the *Methods* section of the main text) and are here averaged over 1881-1910 so as to maximize the overlap with the early industrial era. TX anomalies are referenced to the 1881-1910 climatology (daily normals), over a regular 1×1-degree grid. Black shadings denote areas where data coverage is scarce (less than 50% in the given period for each grid point). As a matter of example, also shown (right panels) are daily TX distributions from observations (1881-1910) and models (1851-1900) over three land regions, i.e., the Euro-Mediterranean (EMD) zone (as in Fig.5a of the main text but for the whole year), Central America (CAM), and Australia (AUS). As seen, model representation of true distributions is rather poor, particularly over Australia, where both variability and negative skewness appear exaggerated.
